# Evaluation of the Impacts of Potassium Bicarbonate, *Moringa oleifera* Seed Extract, and *Bacillus subtilis* on Sugar Beet Powdery Mildew

**DOI:** 10.3390/plants11233258

**Published:** 2022-11-27

**Authors:** Baher A. El-Nogoumy, Mohamed A. Salem, Gabr A. El-Kot, Salem Hamden, Mohamed D. Sehsah, Abeer H. Makhlouf, Yasser Nehela

**Affiliations:** 1 Microbiology Department, Faculty of Science, Kafrelsheikh University, Kafr El-Sheikh 33516, Egypt; 2 Department of Chemistry, Faculty of Science & Arts, King Khalid University, Abha 62529, Saudi Arabia; 3 Department of Chemistry, Faculty of Science, Al-Azhar University, Nasr City, Cairo 11284, Egypt; 4 Department of Agricultural Botany, Faculty of Agriculture, Kafrelsheikh University, Kafr El-Sheikh 33516, Egypt; 5 Plant Pathology Research Institute, Agricultural Research Center, Giza 12619, Egypt; 6 Faculty of Agriculture, Minufiya University, Shibin El-Kom 32511, Egypt; 7 Department of Agricultural Botany, Faculty of Agriculture, Tanta University, Tanta 31511, Egypt

**Keywords:** antioxidant, *Bacillus subtilis*, *Erysiphe betae*, *Moringa oleifera*, PAL, potassium bicarbonate, salicylic acid, scanning electron microscope, sugar beet

## Abstract

Powdery mildew disease, caused by *Erysiphe betae*, is one of the most threatening diseases on sugar beet plants worldwide. It causes a great loss in the root yield, sugar percentage, and quality of produced sugar. In the current study, we aimed to evaluate the susceptibility of 25 sugar beet cultivars to infection with powdery mildew disease under Egyptian conditions. Moreover, we evaluated the impacts of three eco-friendly materials, including potassium bicarbonate (KHCO_3_; at 5 and 10 g L^−1^), *Moringa oleifera* seed extract (25 and 50 g L^−1^), and the biocontrol agent, *Bacillus subtilis* (10^8^ cell suspension) against *E. betae* in two successive seasons 2020 and 2021. Our findings showed that there were significant differences between these 25 cultivars in their susceptibility to the disease under study. Using the detached leaves technique in vitro, *B. subtilis* showed strong antifungal activity against *E. betae*. Moreover, both concentrations of KHCO_3_ and moringa seed extract significantly reduced the disease severity. Under field conditions, tested treatments significantly reduced the severity of powdery mildew disease and prevented *E. betae* from producing its conidiophores and conidia. Scanning electron microscope examination of treated leaves demonstrated the presence of the decomposition of fungal hyphae, conidiophores, conidia, and the occurrence of plasmolysis to fungal cells and spores on the surface of the leaves. Furthermore, these treatments greatly improved the percent of sucrose and soluble solids content, as well as the enzymatic activity of peroxidase, polyphenol oxidase, and phenylalanine ammonia-lyase. It is noteworthy that treatment with moringa seed extract gave the best results, followed by potassium bicarbonate, then *B. subtilis* cell suspension. Generally, it is recommended to use the substances used in this research to combat powdery mildew to minimize or prevent the use of chemical fungicides harmful to public health and the environment.

## 1. Introduction

Sugar beet (*Beta vulgaris* L.) is an important crop for sugar production worldwide. It ranks second in sugar production after sugar cane. In Egypt, about 269 thousand hectares are planted annually with the sugar beet crop with an average production of 18.7 tons per feddan [1]. The demand for sugar has increased due to the annual increase in the population. The Egyptian Ministry of Agriculture, represented by the Sugar Crops Council, aims to increase the cultivated areas of the sugar beet crop in the new season 2022–2021 to approximately 302 thousand hectares.

Powdery mildew of sugar beet, caused by *Erysiphe betae*, is a serious disease worldwide that causes a significant reduction in root yield [2]. The phytopathogenic fungus *E. betae* mainly attacks the leaves of sugar beet. Upon severe attack, up to a 22% reduction in root yield, as well as a 13% reduction in sucrose content in roots, has been recorded [3], thus reducing the yield and quality of the harvested crop [4]. It also reduces extractable root crops and sucrose and increases impurity concentrations, requiring higher processing and significant losses [5].

One of the best means of controlling plant diseases, including the powdery mildew of beets, is the production of resistant varieties [6]. However, the production of resistant varieties requires a lot of time, effort, and money and may not completely meet the required specifications [7]. The most traditional management of powdery mildew mainly relies on the use of fungicides [8]. However, the use of fungicides is instrumental in controlling plant diseases, and chemical control is economically costly and environmentally undesirable. In addition to their harmful effects on the environment and public health, the extensive use of fungicides leads to the development of fungicide-resistant strains of phytopathogenic fungi which has limited their use [9].

Accordingly, there is a necessity to search for new fungicides with different active ingredients. However, chemical fungicides cause serious problems in public health and the ecological system, where their ability to cause many diseases to animals and humans has been proven and they can kill beneficial organisms [10]. Several eco-friendly strategies have been developed to manage various diseases as alternative means for these chemical fungicides. Samples enclose environmentally pleasant chemicals such as potassium bicarbonate, commercially named Armicarb^®^, magnesium sulfate, copper sulfate, and potassium oxide [11,12], plant oils, and plant extracts, such as *Moringa oleifera* plant excerpt [12,13], or the use of microorganisms such as *Bacillus megaterium* and *Trichoderma album*, whether as cultural filtrates or as a suspension [12,14,15,16,17]. At least eighty years ago, Marloth demonstrated the fungal activity of bicarbonate salts [18]. 

Exogenous application of alternative disease management means may affect the biochemical and physiological traits within treated plants under abiotic stress [19,20,21] and biotic challenges such as viral [22], bacterial [23], and fungal [24,25,26] phytopathogens. The major biochemical changes are via the induction of enzymatic and nonenzymatic antioxidant defense machinery [24,25,27,28]. Enzymatic antioxidant defense machinery mainly relies on the activity of some enzymes such as polyphenol oxidase, phenylalanine, ammonia-lyase, peroxidase, and other enzymes [12,14,29]. The scanning electron microscope has been used by many researchers, including Jackowiak [30] and Sehsah [12], to examine the effect of the use of some materials on the growth of pathogens and their fruit structures. 

Previously, bicarbonate salts, particularly sodium and potassium bicarbonate, have been proposed as alternative strategies to control several plant pathogens such as *Venturia inequalis* [11,31], *Pencillium italicum* and *P. digitatuim* [18], *Diplocarpon rosae, Sphaerotheca pannosa* var. *rosae* [32], and *Sphaerotheca fuliginea* [33]. Likewise, products of *M. oleifera* (crude extracts and essential oil) showed strong antifungal activity against some plant pathogenic fungi [13,34,35,36,37]. Previously, we tested the efficacy of *Bacillus subtilis*, *M. oleifera* extract, and potassium bicarbonate on the necrotrophic phytopathogenic fungus *Cercospora beticola*, the causal agent of Cercospora leaf spot on sugar beet [12]. Our previous findings showed that the three proposed treatments significantly enhanced the total soluble solids (TSS) contents and sucrose percentage of treated sugar beet plants, as well as the enzymatic activity of polyphenol oxidase, peroxidase, and phenylalanine ammonia-lyase [12]. However, to the best of our knowledge, the potential effect(s) of *B. subtilis*, *M. oleifera* extract, and potassium bicarbonate on the biotrophic fungus *Erysiphe betae*, the causal agent of sugar beet powdery mildew have been poorly studied. 

Moreover, although the potential effects of Moringa extract on the physio-biochemical attributes of environmentally stressed treated plants have been well-studied [38,39,40,41], its physiological roles with plant response to biotic stress, such as pathogen infection, are poorly studied. For instance, exogenous applications of moringa leaf extract significantly increased the Chlorophyll a, chlorophyll b, carotenoids, and total photosynthetic pigments in stressed common bean leaves [38]. Likewise, it increased the chlorophyll fluorescence (*F_v_/F_m_* and PI), photosynthetic pigments, relative water content (RWC%), and membrane stability index (MSI%) of squash (*Cucurbita pepo*) plants exposed to salt and drought stresses [39]. Additionally, the application of Moringa extract increased the content of chlorophyll a, chlorophyll b, and carotenoids in maize (*Zea mays*) under heat stress [40] and rice (*Oryza sativa*) under drought stress [41]. In terms of plant diseases, soil amendment using Moringa extract significantly reduced the leaf litter, improved the chlorophyll content, and enhanced the response of tomato plants against the soil-borne pathogen, *Fusarium oxysporum f. *sp.* lycopersici,* the causal agent of Fusarium wilt disease [42]. 

In the current study, we aimed to find efficient, economic, and eco-friendly alternatives that could replace chemical fungicides partially or entirely. For this reason, we first evaluated the susceptibility of 25 sugar beet cultivars to infection with the powdery mildew disease under Egyptian conditions. Additionally, we evaluated the impacts of three eco-friendly materials, including potassium bicarbonate, *M. oleifera* seed extract, and the cell suspension of the biocontrol agent, *B. subtilis* on the disease severity, soluble solid contents (TSS), sucrose percentage, and enzymatic activity of peroxidase (POX), polyphenol oxidase (PPO), and phenylalanine ammonia-lyase (PAL) in powdery mildew-infected sugar beet plants during two successive seasons in 2020 and 2021. Moreover, to better understand the potential mechanism(s) by which the tested treatments affected the fungal pathogen, fungal morphological characteristics from the powdery mildew spots on treated plants were examined using scanning electron microscopy (SEM).

## 2. Results

### 2.1. Identification and Pathogenicity of E. betae

The phytopathogenic fungus used in this study was identified based on its morphological and microscopical characteristics and described as *E. betae*. To confirm the pathogenicity of this causal agent and to evaluate the susceptibility of twenty-five sugar beet cultivars, ninety-day old plants were artificially inoculated using conidia of *E. betae*. One-week post-inoculation, the disease severity of powdery mildew in the first season ranged between 2.33 and 70.00% and between 1.33 and 41.66% in the second season. 

**Figure 1:**
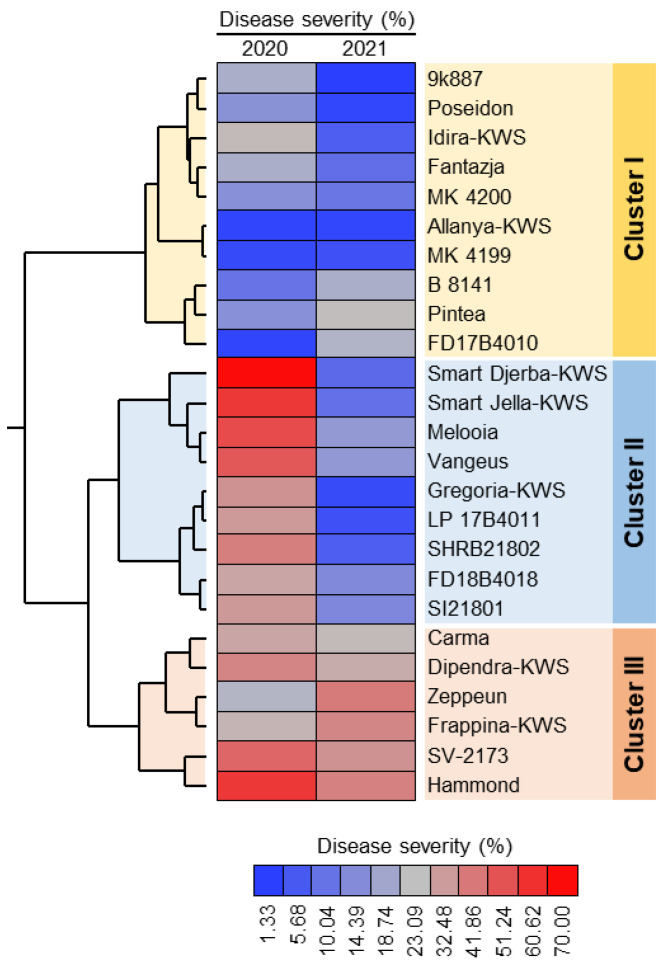
Two-way hierarchical cluster analysis (HCA) of the reaction of 25 sugar beet cultivars to powdery mildew disease caused by *Erysiphe betae* under greenhouse conditions during the 2020 and 2021 seasons. The data presented are the means of three biological replicates (*n* = 3). Rows represent different cultivars, whereas columns represent disease severity (%). Heatmap was colored based on the disease severity percentages where the red color represents a higher disease severity (%) and the blue color represents a lower disease severity (%). See the scale at the bottom of the heatmap. The experiment was repeated twice with similar results.

Two-way hierarchical cluster analysis (HCA) showed that the 25 cultivars were clustered into three main clusters. Cluster-I contained 10 cultivars that were least susceptible to powdery mildew infection including 9k887, Poseidon, Idira-KWS, Fantazja, MK 4200, Allanya-KWS, MK 4199, B 8141, Pintea, and FD17B4010. Cluster-II contained 10 cultivars that were susceptible to powdery mildew disease during 2020 but showed more tolerance during the 2021 season (Figure 1). These nine cultivars included Smart Djerba-KWS, Smart Jella-KWS, Melooia, Vangeus, Gregoria-KWS, LP 17B4011, SHRB21802, FD18B4018, and SI21801. On the other hand, Cluster-III contained only six cultivars that were susceptible and had higher disease severity during both seasons, including Carma, Dipendra-KWS, Zeppeun, Frappina-KWS, SV-2173, and Hammond (Figure 1).

It Is worth mentioning that both Allanya-KWS and FD17B4010 were the least sensitive cultivars (disease severity = 2.33%) during the 2020 season, whereas 9k887 was the least sensitive cultivar (disease severity = 1.33%) during the 2021 season. On the other hand, Smart Djerba-KWS was the most susceptible cultivar (disease severity = 70.00%) during the 2020 season and Zeppeun (disease severity = 41.22%) in the second season.

### 2.2. Efficacy of Different Treatments against Sugar Beet Powdery Mildew Disease under Field Conditions

The efficacy of foliar application of tested treatments included KHCO_3_ (5 g L^−1^), KHCO_3_ (10 g L^−1^), Moringa seed extract (25 mL L^−1^), Moringa seed extract (50 mL L^−1^), *B. subtilis* (1 × 10^8^ cell mL^−1^)*,* Amisto fungicide (azoxystrobin; 1 mL L^−1^), or just sprayed with water (mock control) against sugar beet powdery mildew disease was evaluated under field conditions. Interestingly, all tested treatments significantly decreased the severity of powdery mildew compared to mock-treated control during the 2020 and 2021 seasons (Table 1). The most efficient treatments were the foliar application of moringa seeds extract at 50 mL L^−1^ and *B. subtilis* cell suspension in both tested seasons (Table 1). KHCO_3_ at 10 g L^−1^ followed these treatments in controlling powdery mildew on sugar beet and all of them were similar to Amisto fungicide treatment.

### 2.3. Effect of the Used Treatments on Soluble Solids Contents and Sucrose Percentage of Powdery Mildew-Infected Sugar Beet Plants 

**Figure 2:**
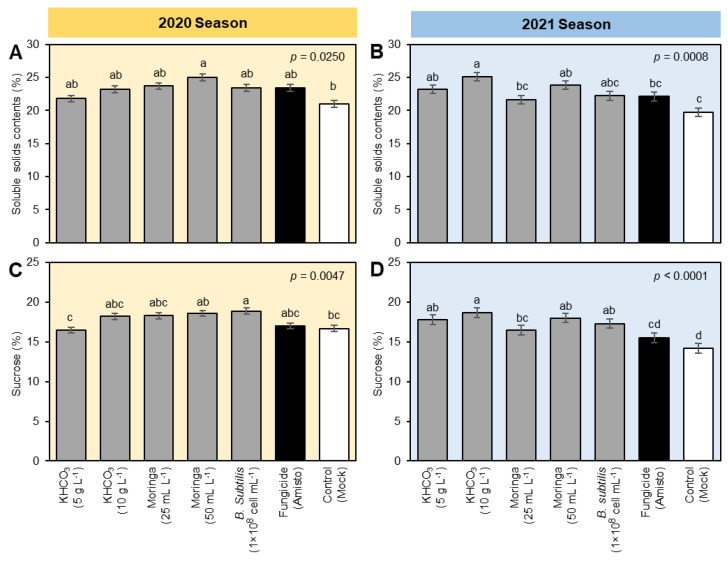
Effect of different treatments on the quality indices of sugar beet plants infected with powdery mildew disease caused by *Erysiphe betae* under field conditions during the 2020 and 2021 seasons. (**A**,**B**) Soluble solid contents (%) during the 2020 and 2021 seasons, respectively, and (**C**,**D**) Sucrose (%) during the 2020 and 2021 seasons, respectively. The data presented are the means ± standard deviation (mean ± SD) of three biological replicates (*n* = 3). Different letters signify statistically significant differences between treatments according to Tukey’s HSD test (*p* ≤ 0.05).

To better understand the potential effect(s) of tested treatments on the economic value and quality indices of treated sugar beet, soluble solids contents (SSC; %) and sucrose percentage were determined in the fresh root of sugar beet (Figure 2). Briefly, soluble solid content (%) was significantly increased in all tested treatments compared to the mock-control with the superiority of Moringa seed extract (50 mL L^−1^) during the 2020 season (Figure 2A) and the application of KHCO_3_ (10 g L^−1^) during the 2021 (Figure 2B) season. Likewise, the sucrose percentage was significantly increased due to the foliar application of all tested treatments compared with both negative (mock-treated) and positive (fungicide-treated) controls. It is worth mentioning that during the 2020 season, application of *B. subtilis* cell suspension showed the highest sucrose percentage, followed by Moringa seed extract (50 mL L^−1^), Moringa seed extract (25 mL L^−1^), and KHCO_3_ (10 g L^−1^) which were similar to Amisto fungicide (Figure 2C). In the second season, KHCO_3_ (10 g L^−1^) had the highest sucrose percentage, followed by Moringa seed extract (50 mL L^−1^), KHCO_3_ (5 g L^−1^), and *B. subtilis* (1 × 10^8^ cell mL^−1^) which were significantly higher than both controls (Figure 2D).

### 2.4. Effect of the Used Treatments on the Activity of Peroxidase (POX), Polyphenol Oxidase (PPO), and Phenylalanine Ammonia-Lyase (PAL) of Powdery Mildew-Infected Sugar Beet Plants

To better understand the biochemical mechanisms of tested treatments, their effect on two main components of the enzymatic antioxidant machinery including peroxidase (POX; Table 2) and polyphenol oxidase (PPO; Table 2) were evaluated. Generally, all tested treatments positively affected the enzymatic activity of POX and PPO in treated leaves of sugar beet with a notable peak at 5 days post first spray. Interestingly, foliar application of moringa seed extract (25 mL L^−1^) or *B. subtilis* cell suspension had the highest POX (Table 2) and PPO (Table 2) activities at 5 days post first spray. 

Likewise, the effect of tested treatments on the activity of phenylalanine ammonia-lyase (PAL; a key enzyme in the salicylic acid (SA) biosynthesis pathway) was also evaluated (Table 2). The enzymatic activity of PAL was incrementally enlarged over time with its highest activity at 15 days post first spray (5 days post second spray). Although there were no significant differences among all treatments at the beginning of the experiment (0 days post first spray), foliar application of moringa seed extract (50 mL L^−1^), moringa seed extract (25 mL L^−1^), or *B. subtilis* cell suspension had the highest PAL activity at 5- and 15-days post first spray (Table 2).

### 2.5. Correlation Analysis between Disease Severity, Soluble Solids, Sucrose Contents, and Antioxidant Enzymes of Powdery Mildew-Infected Sugar Beet Plants

**Figure 3:**
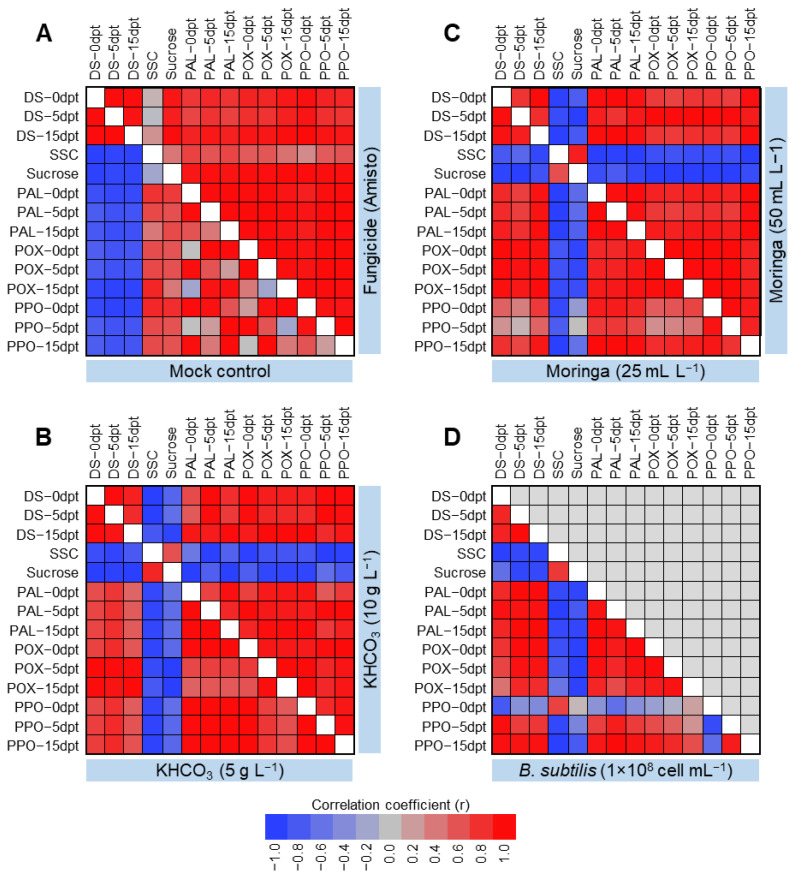
Correlation analysis between disease severity, soluble solids and sucrose contents, and antioxidant enzymes assessed in powdery mildew-infected sugar beet plants. (**A**) Mock and fungicide (Amisto) controls, (**B**) KHCO_3_ (5 vs. 10 g L^−1^), (**C**) Moringa seeds extract (25 vs. 50 mL L^−1^), and (**D**) *B. subtilis* (1 × 10^8^ cell mL^−1^). Three biological replicates were used (*n* = 3). DS: Disease severity (%), SSC: Soluble solids contents (%), POX: Peroxidase activity, PPO: Polyphenol oxidase activity, PAL: Phenylalanine ammonia-lyase activity, and dpt: days post 1st treatment.

The correlation coefficient (r) between disease severity, quality traits (i.e., soluble solids content (SSC) and sucrose percentage), enzymatic activity (i.e., peroxidase activity (POX), polyphenol oxidase activity (PPO), and phenylalanine ammonia-lyase activity (PAL)) of powdery mildew-infected sugar beet plants were determined. In mock-treated plants, disease severity was negatively correlated with all other parameters (Figure 3A). However, treatment with Amisto fungicide significantly reversed this correlation (Figure 3A). In KHCO_3_-treated plants, the disease severity was highly and negatively correlated with the quality traits (SSC and sucrose) but positively correlated with the enzymatic activities of POX, PPO, and PAL (Figure 3B). it is worth mentioning that the high concentration of KHCO_3_ (10 g L^−1^) significantly strengthened this relationship. Likewise, in Moringa-treated plants, the disease severity (%) was positively correlated with the enzymatic activities of POX, PPO, and PAL at 0, 5, and 15 dpt, but negatively correlated with SSC and sucrose percentage (Figure 3C). The foliar application of Moringa seed extracts (50 mL L^−1^) noticeably enhanced this relationship. In *B. subtilis*-treated plants, disease severity was positively correlated with the enzymatic activities of POX, PPO, and PAL at all studied time points, except with PPO at 0 dpt (Figure 3D). On the other hand, disease severity (%) was negatively correlated with quality traits (i.e., SSC and sucrose percentage) and PPO activity at 0 dpt. 

### 2.6. Scanning Electron Microscopy (SEM) Examination of the Interaction among the Most Promising Treatments and E. betae on Leaves of Sugar Beet 

**Figure 4:**
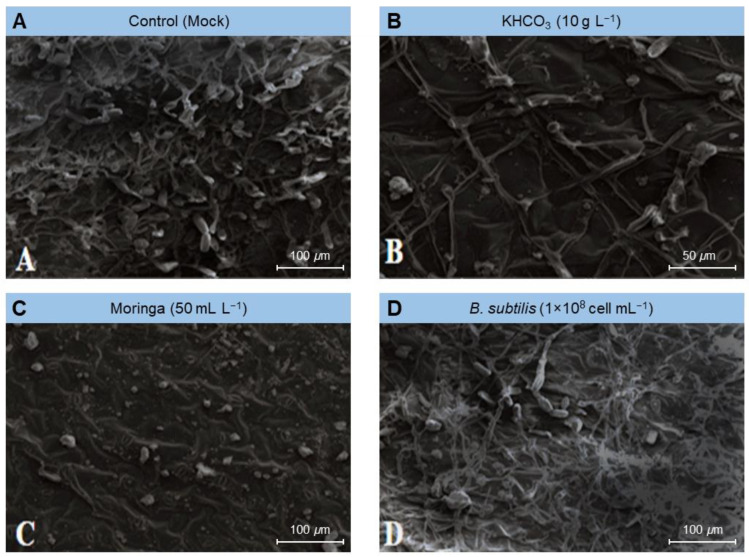
Effect of the most promising treatments on the mycelium density and conidia formation of *Erysiphe betae*, the causal agent of powdery mildew on sugar beet using scanning electron microscopy (SEM). (**A**) mock-treated plants (tap water), (**B**) plants treated with 10 g L^−1^ Potassium bicarbonate, (**C**) plants treated with 50 mL L^−1^ Moringa seeds extract, and (**D**) plants treated with a cell suspension of *Bacillus subtilis* (1 × 10^8^ cell mL^−1^).

To gain greater knowledge about the potential mechanism(s) by which the tested treatments affected the fungal morphology, several fungal morphological characteristics were also examined from the powdery mildew spots on treated plants compared to those from mock-treated ones (control) using scanning electron microscopy (SEM). The examined fungal morphological characteristics included growth, conidiophores and conidia density, and mycelium and conidia decomposition. Our SEM findings showed that the foliar application of KHCO_3_ (10 g L^−1^; Figure 4B), Moringa seed extract (50 mL L^−1^; Figure 4C), or *B. subtilis* (1 × 10^8^ cell mL^−1^; Figure 4D) significantly decreased the density of the fungal mycelium, especially on leaves treated with Moringa extract (Figure 4C) and potassium bicarbonate (Figure 4B), followed by the suspension of Bacillus cells (Figure 4D).

**Figure 5:**
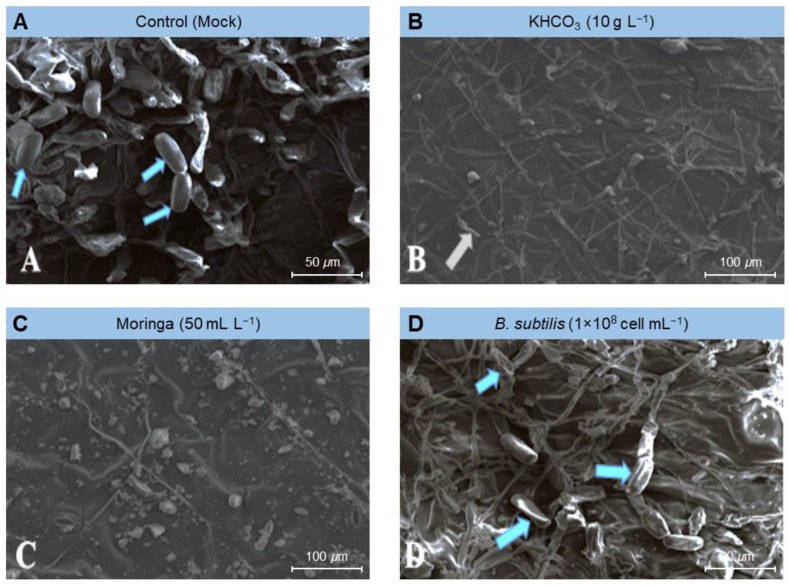
Effect of the most promising treatments on the morphology and number of conidia formed by *Erysiphe betae*, the causal agent of powdery mildew on sugar beet using scanning electron microscopy (SEM). (**A**) mock-treated plants (tap water), (**B**) plants treated with 10 g L^−1^ potassium bicarbonate, (**C**) plants treated with 50 mL L^−1^ Moringa seeds extract, and (**D**) plants treated with a cell suspension of *Bacillus subtilis* (1 × 10^8^ cell mL^−1^).

**Figure 6:**
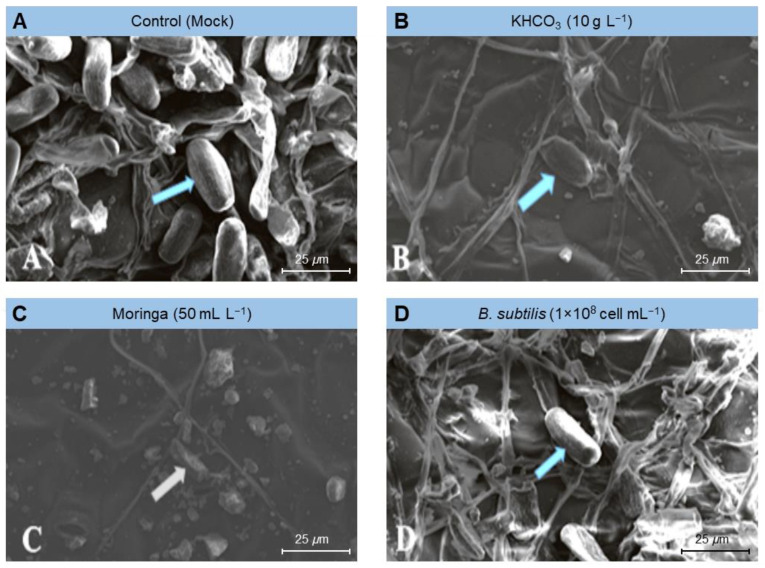
Effect of the most promising treatments on the decomposition of mycelium and conidia of *Erysiphe betae*, the causal agent of powdery mildew on sugar beet using scanning electron microscopy (SEM). (**A**) mock-treated plants (tap water), (**B**) plants treated with 10 g L^−1^ potassium bicarbonate, (**C**) plants treated with 50 mL L^−1^ Moringa seeds extract, and (**D**) plants treated with a cell suspension of *Bacillus subtilis* (1 × 10^8^ cell mL^−1^).

Similarly, the above-mentioned treatments markedly dropped the number of conidia formed by the *E. betae* and diminished the capacity of the phytopathogenic fungus to form conidiophores and conidia (Figure 5). Moreover, it caused plasmolysis and decomposition of the mycelium of *E. betae* and its conidia. Interestingly, the conidia on treated leaves appeared in an incomplete state, and their construction appeared in a distorted form (Figure 5 and Figure 6).

## 3. Discussion

In the current study, we evaluated the utilization of *B. subtilis* cell suspension as a biocontrol strategy against *E. betae*, the causal agent of powdery mildew of sugar beet along with another four eco-friendly strategies including two concentrations of potassium bicarbonate and two concentrations of *M. oleifera* seeds extract. 

Initially, the susceptibility of 25 plant specimens was evaluated under the artificial inoculation of *E. betae*. These varieties showed great variation in the extent of their susceptibility to powdery mildew caused by the fungus *E. betae*, after artificially infecting them by dispersing the fungus spores from old leaves of infection on the leaves of resident varieties plants. These results are in agreement with the previous research that was performed on powdery mildew, as it indicated a difference in the susceptibility of plant varieties to powdery mildew due to the genetic differences between those varieties [16,17].

Allanya-KWS, 9k887, and FD17B4010 were the least susceptible (most resistant) cultivars to the infection with the fungus *E. betae* and could be generalized in future cultivation. On the other hand, Smart Djerba-KWS and Zeppeun were the most susceptible cultivars which should be eliminated and not be cultivated under Egyptian conditions. Previous studies assessed the resistance of some sugar beet varieties to powdery mildew using crossed immune-electrophoresis technique [43,44]. They explained that there are several *E. betae* resistance genes. The variety containing these genes appears to be more resistant to the disease than the variety lacking these genes.

Chemical control, despite containing substances that kill beneficial microbes and negatively affect public health and the environment, is still the first choice for combating plant diseases in general and powdery mildew in particular. Therefore, there will always be a need to search for safe alternatives to fungicides and at the same time give results that are close or similar and perhaps better than those agrochemicals and less harmful to public health and the environment. Biological control, whether by using bioagents, oils, plant extracts, or other eco-friendly materials, is considered one of the most promising options that attract great attention in this field. 

Our findings showed that foliar application of *B. subtilis* cell suspension (1 × 10^8^ cell mL^−1^) significantly reduced the disease severity of sugar beet powdery mildew disease under field conditions. Moreover, it increased the sucrose percentage, and the enzymatic activity of POX, PPO, and PAL but decreased the density of the fungal mycelium on treated leaves. These results are in agreement with those of Bakeer et al. [45] who recently reported that the commercial biocide Biobac 50% WP (*B. subtilis*; 1 × 10^9^ cell g^−1^) showed significant antifungal activity against *E. heraclei*, the causal agent of powdery mildew of parsley *under* in vitro conditions. We suggest that the antifungal activity of biological agents, such as *B. subtilis,* might be due to their ability to biosynthesize and produce several metabolites such as antibiotics, hydrolytic enzymes, and toxic compounds, these metabolites could cause degradation and lyse to the cell wall of fungal mycelium and spores or inhibit some enzymes that are essential for spore germination [45]. However, further studies are required to deeply analyze the chemical composition of extracellular metabolites of *B. subtilis.*

Several studies have attributed the inhibition of the growth of various pathogens by *Bacillus subtilis* to its ability to produce some antibiotics such as mycoseptillin and trippilin [14,46,47]. Others have demonstrated that there are other ways for *Bacillus* sp. to inhibit the growth of phytopathogens or reduce the competitiveness with other microorganisms, such as the production of antibiotics or vital growth enzymes. Antibiotics, enzymes, and peptide antibiotics are substances with amphiphilic membrane activity and surfactants for pathogens. In the current study, and under field conditions, *B. subtilis* showed a significant reduction in the severity of powdery mildew disease. This is due to its high ability to spread rapidly on the surface of plant leaves and prevent pathogenic germs from reaching the natural openings, thus preventing infection. 

Biocontrol agents also have the advantage of being able to compete intensely for oxygen and nutrients on the surface of leaves, prohibiting pathogens of access them, and starving them [48,49]. Examination using scanning electron microscopy (SEM) also demonstrated that *Bacillus subtilis* induced plasmolysis and lysis of conidiophores and conidia of *E. betae.* This finding is in line with the demonstration from several similar cases with phytopathogenic fungi, reported previously, which indicated that *B. subtilis* secretes fungal cell-degrading enzymes including b-1, 3-gluconase, and protease [12,50].

Other eco-friendly alternatives to chemical fungicides use beneficial chemicals and or plant extracts. For instance, potassium bicarbonate (KHCO_3_) is a beneficial eco-friendly chemical for plants; however, our knowledge about its role in plant response to biotic stressors is still limited. Likewise, antifungal activities of plant extracts, such as extracts derived from *M. oleifera,* have been reported against several phytopathogens [34,45,51]. Nevertheless, these fungistatic activities were due to the utilization of *M. oleifera* essential oil and seed extract, but not crude leaf extract [35]. Herein, we tested the effectiveness of two different concentrations of KHCO_3_ (5 and 10 g L^−1^) and two concentrations of moringa seed extract to control of powdery mildew of sugar beet. Our finding showed that both Moringa seed extract and KHCO_3_ caused a significant decrease in the growth of *E. betae* to varying degrees. Furthermore, they showed a great ability to combat the powdery mildew of sugar beet under field conditions. Additionally, certain concentrations of Moringa seed extract and KHCO_3_ significantly increased SSC and sucrose percentage, and the enzymatic activity of POX, PPO, and PAL.

Scanning electron microscope (SEM) examination of samples treated with potassium bicarbonate showed that the fungal structures were decomposed and their inability to produce both conidia and conidiophores. Previous research suggests that potassium bicarbonate can be used to combat certain plant diseases such as powdery mildew, apple scorch spots, and scab [11,31], grapevine powdery mildew [52], powdery mildew on cucumber [33], Cercospora leaf spot of sugar beet [12] and black spot and powdery mildew of roses [32]. We suggest that the action of potassium bicarbonate is associated with the occurrence of a disturbance in the osmotic pressure and the bio-carbon/bicarbonate ion balance of sensible fungi, and the pH. Bicarbonate prevents the development of fungi and their ability to sporulate through contact with fungi in an aqueous solution. It is therefore believed that the possibility of the emergence of resistant strains of bicarbonate is low due to the multiplicity of its modes of action [31,32]. 

Similarly, in this study, whether in the laboratory or the field, the two concentrations used of Moringa seed extract (25 and 50 g L^−1^) significantly reduced the linear growth of *E. betae* and the severity of powdery mildew disease. The antimicrobial activity of *M. oleifera* extract was evaluated previously against *Fusarium solani*, *Pasturella multocida*, *Staphylococcus aureus*, *Escherichia coli*, and *Rhizopus solani* strains [36]. Previous reports on the antibiotic principle results of *M. oleifera* seeds through their purification, clarification, and antimicrobial properties, as well as on the antibiotic substance of *M. oleifera* roots have been demonstrated [13,53]. Several studies have proven that certain plant extracts contain many toxins and fungi inhibitors that negatively affect the growth of pathogens [54,55,56]. Phytochemical analysis revealed the presence of glycosides, alkaloids, triterpenoids, flavonoids, steroids, and tannins [37]. Seed extracts of moringa also contain organic compounds and pigments such as flavonoids, carotenoids, niacin, isothiocyanates, minerals, sterols, and glucosinolates, all of which are accountable for the fashioning of antioxidants [36,57]. 

To better understand the positive results of tested treatments to control powdery mildew of sugar beet, the effect of the studied treatments on the activity of some oxidation enzymes, such as POX and PPO, and the key enzyme in SA biosynthesis pathway, PAL, was evaluated, and its action was related to stimulating plants to resist pathogens [12,58,59]. Our findings from the current study proved that foliar application of sugar beet plants with KHCO_3_, Moringa seed extract, or *B. subtilis* (1 × 10^8^ cell mL^−1^) led to a significant improvement in the activity of these enzymes. This improvement in the activity of defense enzymes helps to reveal additional explanations for the ability of the treatments to reduce the severity of the pathogen under study, in addition to the possibility of improving some of the good qualities of sugar beet, such as the content of soluble solids and sucrose percentage.

## 4. Materials and Methods

### 4.1. Pathogen Identification, Pathogenicity, and Susceptibility of Sugar Beet Cultivars

In the current study, the phytopathogenic fungus was identified according to Barnet and Hunter [60]. Subsequently, a pathogenicity test was carried out in 35 cm diameter pots under greenhouse conditions. Sandy-loam soil (1:2 *w*/*w*; pH = 6.7, available P_2_O_5_ = 284 mg Kg^−1^, available K_2_O = 252 mg Kg^−1^, N = 15 g Kg^−1^, and humus = 17.8 g Kg^−1^) was used throughout the study. Twenty-five sugar beet varieties (namely 9k887, Allanya-KWS, B 8141, Carma, Dipendra-KWS, Fantazja, FD17B4010, FD18B4018, Frappina-KWS, Gregoria-KWS, Hammond, Idira-KWS, LP 17b4011, Melooia, MK 4199, MK 4200, Pintea, Poseidon, SHRB21802, SI21801, Smart Djerba KWS, Smart Jella-KWS, SV-2173, Vangeus, and Zeppeun) were utilized in this investigation to test the pathogenicity of the causal agent and to evaluate the susceptibility of these varieties to infection with powdery mildew. To test the sensitivity of these varieties to powdery mildew, they were artificially infected by applying conidia for *E. betae* on the leaves of those plants from aged, infected leaves. The percent of disease severity was documented according to Shane and Teng [61] after 100 days from sowing. 

### 4.2. Treatments

In addition to the negative control (mock-treated plants), Amisto (Azoxystrobin) 25% SC, at its recommended rate (1 mL L^−1^), was used as positive control throughout the study. Potassium bicarbonate (KHCO_3_; Al-Gomhoria Company for Chemicals and Glasses, Cairo, Egypt) was used at the rate of 5 or 10 g L^−1^. *Bacillus subtilis* was isolated from nourishing sugar beet leaves and identified according to Bergey’s Manual of Systematic Bacteriology [62] and used as a bioagent at 1 × 10^8^ cell mL^−1^ in this study. The seed extract of *Moringa oleifera* was applied at 25- or 50-mL L^−1^. All experiments were carried out using a completely randomized design (CRD) with three biological replicates per treatment. 

### 4.3. Evaluation of the Tested Treatments against Erysiphe betae under Field Conditions

The susceptible cultivar ‘Pleno’ was used throughout the field experiments. All experiments were carried out at the Research Experimental Farm of Plant Pathology Research Institute, Sakha Station, Kafrelsheikh, Egypt using a completely randomized design with three biological replicates during two successive seasons (2020 and 2021). Each biological replicate was composed of six rows with 900 cm length and 60 cm width. Each row contained 45 hills 20 cm apart. All recommended cultural practices were performed at the proper time. Plants were sprayed three times, with 10 day intervals between them, with one of the following treatments, KHCO_3_ (5 g L^−1^), KHCO_3_ (10 g L^−1^), Moringa seed extract (25 mL L^−1^), Moringa seed extract (50 mL L^−1^), *B. subtilis* (1 × 10^8^ cell mL^−1^)*,* Amisto fungicide (1 mL L^−1^), or just sprayed with water (Mock control). The first spray was after approximately 90 days of cultivation when disease symptoms were detected. 

#### 4.3.1. Disease Severity

Disease severity was estimated after 10 days of the first treatment (initial disease severity) and twice more with ten days intervals. In other words, the last appraisal took place 30 days after the first application (the final disease severity). The powdery mildew disease severity was estimated according to McGrath and others [63], by calculating observable sporulating mildew colonies on both adaxial and abaxial textures per leaf. Briefly, five old leaves per plant were examined for five plants in each plot (i.e., 25 leaves/treatment). Assessments were also made on fully expanded leaves from the middle and upper thirds of a plant. Data from all three age classes of leaves were averaged concurrently. The initial disease severity ranged widely under field conditions, due to the presence of powdery mildew colonies on sugar beet leaves before treatment. The experiment was carried out using a completely randomized design (CRD) with three biological replicates per treatment. The corrected final disease severity (A) was calculated as the result of corrected disease severity (*CDS*) according to Equation (1) as described by Kamel [64] as follows:

(1) $$CDS\;=\;\frac{L}{M}\times N$$

where *CDS*: Corrected disease severity, *L*: the initial disease severity of treatment, *M*: the initial disease severity of the check (control), and *N*: the final disease severity of the control.

#### 4.3.2. The Soluble Solid Content and Sucrose Percentage

Soluble solid content (SSC; %) was determined in the fresh root of sugar beet using a hand refractometer according to McGinnis [65]. Moreover, the sucrose percentage was estimated according to AOAC [66]. 

#### 4.3.3. Enzymatic Activity

Generally, leaf fresh samples were collected for the enzymatic activity assays at 0, 5, and 15 days post the first treatment (dpt). Briefly, one gram of leaf tissue was ground in 2 mL of 0.1 M sodium phosphate buffer (pH 7.1) using a porcelain mortar. Subsequently, samples were centrifuged at 3000 rpm for 20 min at 6 °C and the supernatants were collected and considered as a crude enzyme extract. Peroxidase (POX) activity was determined according to the method of Allam et al. [67] by estimating the oxidation of pyrogallol to pyrogalline in the presence of hydrogen peroxide. The enzymatic activity of POX was calculated by following the differences in absorbance at 425 nm every 1 min for five minutes using Beckman Spectrophotometer Du^®^ 7400(Beckman Coulter Inc., Fullerton, CA, USA). Likewise, the enzymatic activity of polyphenol oxidase (PPO) was spectrophotometrically determined according to Maxwell and Batman [68]. Briefly, PPO activity was calculated by following the changes in absorbance at 495 nm every 1 min for five minutes using the same spectrophotometer mentioned above. Furthermore, the enzymatic activity of phenylalanine ammonia-lyase (PAL) was determined according to the method of Zucker [69]. Briefly, PAL activity was estimated in acetone powder prepared from leaves, using 0.75 gm acetone powder suspended in sodium borate buffer (pH 8.8). The reaction mixture included 0.5 mL enzyme preparation, 1.5 mL borate buffer 0.2 M (pH 8.8), 1 mL of 1% phenylalanine, and 2.5 mL deionized water. One mL of deionized water was added instead of phenylalanine as a blank. The mixture was incubated at 40 °C for one hour. The reaction was stopped by adding 0.5 mL of 5N HCl to each tube. The enzyme activity was measured at 290 nm and expressed as μg *t*-cinnamic acid g^−1^ FW. The experiment was carried out using a completely randomized design (CRD) with three biological replicates per treatment.

### 4.4. Scanning Electron Microscopy (SEM) Examination 

The effectiveness of the used treatments on the formation of conidia and spores, as well as the growth of *E. betae* on sugar beet leaves, was investigated using scanning electron microscopy (SEM) according to Manzali et al. [70]. Interaction sites (spots) were marked and disc blocks of 1 cm^2^ were taken for SEM using Jeol Scanning Electron Microscope model JSM-5500lv (JEOL Ltd., Akishima, Tokyo, Japan) at Electron Microscope Unit, Nanotechnology Institute, Kafrelsheikh University. Samples, illustrating the interaction region, were appointed with osmium oxide, and then dehydrated, using a serial dilution of ethyl alcohol and then finally acetone. Processed samples were then dried, using a critical point drier (EMS 850; Electron Microscopy Sciences [EMS], Hatfield, PA, USA), coated with gold using a sputter coater (EMS 550; EMS, Hatfield, PA, USA), then the samples were investigated using an SEM (Jeol 100cx–11 ASID—4D; JEOL Ltd., Akishima, Tokyo, Japan).

### 4.5. Statistical Analysis

All experiments were carried out using a completely randomized design with three biological replicates during two successive seasons (2020 and 2021). All data were statistically analyzed using the analysis of variance (ANOVA), followed by the Tukey–Kramer honestly significant difference test (Tukey HSD, *p* ≤ 0.05) as a post hoc analysis for pairwise comparisons. Moreover, hierarchical cluster analysis (HCA) was used to better understand the variations in susceptibility of different cultivars. Finally, correlation analysis was conducted to evaluate the relationships between disease severity, quality traits (i.e., SSC and sucrose percentage), and enzymatic activity (i.e., POX, PPO, and PAL) of powdery mildew-infected sugar beet plants. Correlation coefficients (r) are presented as a heatmap.

## 5. Conclusions

Collectively, our findings demonstrated that foliar application of KHCO_3_, Moringa seed extract, or *B. subtilis* cell suspension significantly enhanced sugar beet resilience to the infection with *E. betae*, the causal agent of powdery mildew disease. These treatments are eco-friendly, less expensive, and may replace commercial fungicides totally or partially. Application of KHCO_3_, Moringa seed extract, or *B. subtilis* cell suspension significantly reduced the severity of powdery mildew disease but induced the SSC and sucrose percentage. The protective role(s) of these compounds might be due to the activation of enzymatic antioxidant machinery (as expressed by higher enzymatic activities of POX and PPO) or the enhancement of SA biosynthesis (as expressed by higher enzymatic activities of PAL). Nevertheless, further investigations are required to determine the chemical composition of Moringa seed extract and *B. subtilis* extracellular metabolites and their molecular mechanisms during the *E. betae*–beet interactions.

## Figures and Tables

**Table 1:** Efficacy of different treatments against powdery mildew disease caused by *Erysiphe betae* under field conditions during the 2020 and 2021 seasons ^a^.

Treatments	2020 Season					
	Disease Severity (%)			Efficacy (%)		
	1st Spray	2nd Spray	3rd Spray	1st Spray	2nd Spray	3rd Spray
Control (Mock)	2.52 ± 0.11 a	3.95 ± 1.34 a	5.60 ± 1.13 a	0.00 ± 0.00 g	0.00 ± 0.00 d	0.00 ± 0.00 d
Amisto fungicide (Azoxystrobin)	0.72 ± 0.16 b	0.91 ± 0.16 b	1.50 ± 0.85 b	72.10 ± 1.56 a	76.90 ± 1.27 a	73.20 ± 0.99 a
Potassium bicarbonate (5 g L^−1^)	1.40 ± 0.14 ab	1.70 ± 0.14 b	2.30 ± 0.99 b	44.10 ± 1.27 f	56.40 ± 1.70 c	59.00 ± 0.71 c
Potassium bicarbonate (10 g L^−1^)	1.08 ± 0.18 ab	1.53 ± 0.18 b	2.50 ± 0.85 b	56.15 ± 1.63 d	61.50 ± 1.56 b	55.42 ± 0.96 c
Moringa seeds extract (25 mL L^−1^)	1.31 ± 0.16 ab	1.61 ± 0.16 b	2.00 ± 1.56 b	48.00 ± 1.41 e	58.90 ± 1.27 c	64.30 ± 1.13 b
Moringa seeds extract (50 mL L^−1^)	1.01 ± 0.15 ab	1.21 ± 0.13 b	1.90 ± 0.99 b	60.00 ± 2.83 c	69.20 ± 1.13 b	66.40 ± 0.85 b
*Bacillus subtilis*	0.91 ± 0.13 b	1.32 ± 0.12 b	1.90 ± 0.85 b	64.05 ± 1.48 b	66.60 ± 0.99 b	66.10 ± 1.56 b
*p*-value	<0.0001	<0.0001	=0.0002	<0.0001	<0.0001	<0.0001
	**2021 season**					
	**Disease severity (%)**			**Efficacy (%)**		
	**1st spray**	**2nd spray**	**3rd spray**	**1st spray**	**2nd spray**	**3rd spray**
Control (Mock)	3.60 ± 0.71 a	5.80 ± 1.27 a	8.70 ± 0.99 a	0.00 ± 0.00 e	0.00 ± 0.00 d	0.00 ± 0.00 d
Amisto fungicide (Azoxystrobin)	1.10 ± 0.57 c	1.90 ± 0.99 b	2.50 ± 0.85 b	69.40 ± 0.85 a	67.20 ± 0.85 b	71.30 ± 1.70 b
Potassium bicarbonate (5 g L^−1^)	2.20 ± 1.56 ab	2.70 ± 0.85 b	3.40 ± 1.27 b	38.30 ± 2.40 d	53.40 ± 0.71 c	60.10 ± 0.71 c
Potassium bicarbonate (10 g L^−1^)	1.90 ± 1.27 b	2.60 ± 0.71 b	2.80 ± 1.41 b	47.20 ± 1.56 c	55.20 ± 0.85 b	67.80 ± 0.99 b
Moringa seeds extract (25 mL L^−1^)	2.31 ± 1.26 ab	2.80 ± 1.13 b	3.20 ± 0.99 b	36.10 ± 1.27 d	51.70 ± 1.56 c	63.20 ± 0.85 c
Moringa seeds extract (50 mL L^−1^)	2.20 ± 1.70 ab	2.80 ± 1.41 b	3.00 ± 1.27 b	38.80 ± 1.56 d	60.30 ± 0.99 b	65.50 ± 1.27 b
*Bacillus subtilis*	1.60 ± 0.99 c	2.40 ± 0.85 b	2.80 ± 0.71 b	55.50 ± 1.70 b	58.60 ± 0.71 b	67.80 ± 0.99 b
*p*-value	=0.0080	=0.0005	<0.0001	<0.0001	<0.0001	<0.0001

^a^ The data presented are the means ± standard deviation (mean ± SD) of three biological replicates (*n* = 3). Different letters signify statistically significant differences between treatments according to Tukey’s HSD test (*p* ≤ 0.05).

**Table 2:** Effect of different treatments on the enzymatic activity of peroxidase (POX; antioxidant enzyme), polyphenol oxidase (PPO; antioxidant enzyme), and phenylalanine ammonia-lyase (PAL; SA biosynthetic enzyme) of sugar beet plants infected with powdery mildew disease caused by *Erysiphe betae* under field conditions during the 2020 and 2021 seasons *.

	Peroxidase Activity (Δabs. Min^−1^ g^−1^)			Polyphenol Oxidase Activity (Δabs. Min^−1^ g^−1^)			Phenylalanine Ammonia-Lyase Activity (μg *t*-Cinnamic Acid g^−1^ FW)		
	0 dpt	5 dpt	10 dpt	0 dpt	5 dpt	10 dpt	0 dpt	5 dpt	10 dpt
Control (Mock)	0.034 ± 0.002 ns	0.113 ± 0.006 c	0.054 ± 0.003 d	0.032 ± 0.002 c	0.265 ± 0.013 b	0.215 ± 0.011 c	0.119 ± 0.006 ns	0.214 ± 0.011 c	0.304 ± 0.015 c
Amisto fungicide (Azoxystrobin)	0.034 ± 0.002 ns	0.135 ± 0.007 b	0.122 ± 0.006 cd	0.037 ± 0.002 c	0.263 ± 0.013 b	0.263 ± 0.013 b	0.130 ± 0.007 ns	0.276 ± 0.014 b	0.387 ± 0.019 b
Potassium bicarbonate (5 g L^−1^)	0.048 ± 0.002 ns	0.178 ± 0.009 a	0.189 ± 0.009 a	0.070 ± 0.004 b	0.241 ± 0.012 b	0.340 ± 0.017 a	0.122 ± 0.006 ns	0.203 ± 0.010 c	0.326 ± 0.016 c
Potassium bicarbonate (10 g L^−1^)	0.047 ± 0.002 ns	0.151 ± 0.008 b	0.149 ± 0.007 b	0.071 ± 0.004 b	0.234 ± 0.012 b	0.331 ± 0.017 a	0.123 ± 0.006 ns	0.206 ± 0.010 c	0.331 ± 0.017 c
Moringa seeds extract (25 mL L^−1^)	0.042 ± 0.002 ns	0.186 ± 0.009 a	0.184 ± 0.009 a	0.110 ± 0.006 a	0.368 ± 0.018 a	0.230 ± 0.011 c	0.132 ± 0.007 ns	0.304 ± 0.015 a	0.411 ± 0.021 a
Moringa seeds extract (50 mL L^−1^)	0.027 ± 0.001 ns	0.139 ± 0.007 b	0.145 ± 0.007 b	0.095 ± 0.005 a	0.247 ± 0.012 b	0.231 ± 0.012 c	0.134 ± 0.007 ns	0.309 ± 0.015 a	0.427 ± 0.021 a
*Bacillus subtilis*	0.032 ± 0.002 ns	0.184 ± 0.009 b	0.131 ± 0.007 bc	0.057 ± 0.003 b	0.378 ± 0.019 a	0.291 ± 0.015 b	0.099 ± 0.005 ns	0.337 ± 0.017 a	0.412 ± 0.021 a
*p*-value	=0.0594	<0.0001	<0.0001	<0.0001	<0.0001	<0.0001	<0.0601	<0.0001	<0.0001

* The data presented are the means ± standard deviation (mean ± SD) of three biological replicates (*n* = 3). Different letters signify statistically significant differences between treatments, whereas “ns” signify no significant differences between them according to Tukey’s HSD test (*p* ≤ 0.05). The experiment was repeated twice with similar results. dpt: Days post 1st treatment/spray.

## Data Availability

The data collected and analyzed throughout the present research are available upon request.
